# Is R1 Extended Liver Resection for Locally Advanced Intrahepatic Cholangiocarcinoma Justified? Nodal Status Not Margin Drives Prognosis

**DOI:** 10.1245/s10434-026-20034-3

**Published:** 2026-06-29

**Authors:** Edoardo Maria Muttillo, Daniel Cherqui, Marc-Anthony Chouillard, Nicolas Golse, Oriana Ciacio, Gabriella Pittau, Chady Salloum, Daniel Azoulay, Marc-Antoine Allard, Daniel Pietrasz, René Adam, Eric Vibert, Pascal Hammel, Maïté Lewin, Alina Pascale, Olivier Rosmorduc, Antonio Sa Cunha

**Affiliations:** 1 Henri-Bismuth Hepatobiliary Center, Paul-Brousse Hospital, Assistance Publique-Hôpitaux de Paris, Paris-Saclay University, Villejuif, France; 2 Department of Medical Oncology, Paul-Brousse Hospital, Assistance Publique-Hôpitaux de Paris, Paris-Saclay University, Villejuif, France; 3 Department of Radiology, Paul-Brousse Hospital, Assistance Publique-Hôpitaux de Paris, Paris-Saclay University, Villejuif, France

**Keywords:** Locally advanced intrahepatic cholangiocarcinoma, Extended hepatectomy, R1 resection, Extended liver resection, Advanced liver surgery

## Abstract

**Background and Purpose:**

Surgery for locally advanced intrahepatic cholangiocarcinoma (LAICC) requires extended liver resections, often associated with vascular and/or biliary reconstruction. The benefits of these high-risk operations are still debated. The objective of this study was to analyze short, long-term outcomes, and futility after surgery for LAICC.

**Methods:**

A retrospective single-center study on the 2013–2024 period was conducted. LAICC was defined as mass-forming intrahepatic tumors 5 cm or more in size with hepatic vein/IVC contact or hepatic hilum contact or both and requiring extended liver resection (five segments or more). Futility was defined as deaths within 90 days or recurrence within 6 months of surgery.

**Results:**

39 consecutive patients were analyzed, including (29) 74% women with a median age of 66 years (38–83 years). In 34 (88%) patients, a trisectionectomy (H145678 or H123458) was performed. Major vascular or biliary recontruction was required in 43.5% and 67%, respectively. Overall morbidity was 56%, with severe morbidity occurring in 8 (20%). The 90-day mortality was 5%. Median overall survival and recurrence-free survival was estimated at 58 and 23 months, respectively. Overall rate of futility was 15.4%. N+ has been shown to be the main factor affecting survival (*p* = 0.03 for overall survival and *p* = 0.01 for recurrence-free survival). R1 resection had no impact of overall or recurrence-free survival.

**Conclusions:**

Our study supports an aggressive surgical approach for LAICC. Anticipated R1 resection by necessity should not be considered a contraindication to surgery.

**Supplementary Information:**

The online version contains supplementary material available at https://doi.org/10.1245/s10434-026-20034-3.

Intrahepatic cholangiocarcinoma (ICC) is the second most common primary liver malignancy and is an increasing cause of global cancer mortality.^1^ The worldwide incidence is estimated at approximately 0.95–1.4 cases per 100,000 individuals, with a 5-year overall survival rate of less than 25%.^2^ Currently, liver resection with regional lymphadenectomy is considered the condition for curative treatment, offering 5-year survival rates of up to 35–40% in selected patients.^2^ The disease,frequently asymptomatic in its early phases, commonly presents at an advanced stage, making assessment of resectability particularly challenging. Current consensus on resectability is defined as the possibility of achieving an R0 resection while preserving an adequate future liver remnant.^3^ This definition, however, remains subjective and surgeon/center-dependent. Consequently, the determination of resectability often varies across institutions, resulting in heterogeneous surgical strategies, and large multicentric studies usually lack granularity. Centrally located lesions with vascular contact at the hepatocaval confluence, the hepatic hilum, or both remain a major subject of debate owing to the complexity of surgical management and unclear prognostic implications. The European Association for the Study of the Liver (EASL) guidelines acknowledge the complexity of these cases, and in some scenarios, consider them potential contraindications to surgical resection owing to high morbidity and mortality.^4^ Similarly, long-term outcomes remain unclear: recurrence occurs in over 60% of cases, and 5-year overall survival rarely exceeds 30%, raising questions about the true benefit of extensive resections.^5^ This study aims to evaluate the perioperative and oncologic outcomes of patients with locally advanced ICC (LAICC) who underwent extended liver resection (ELR) at a high-volume tertiary hepatobiliary center over the past decade.

## Methods

**Fig. 1:**
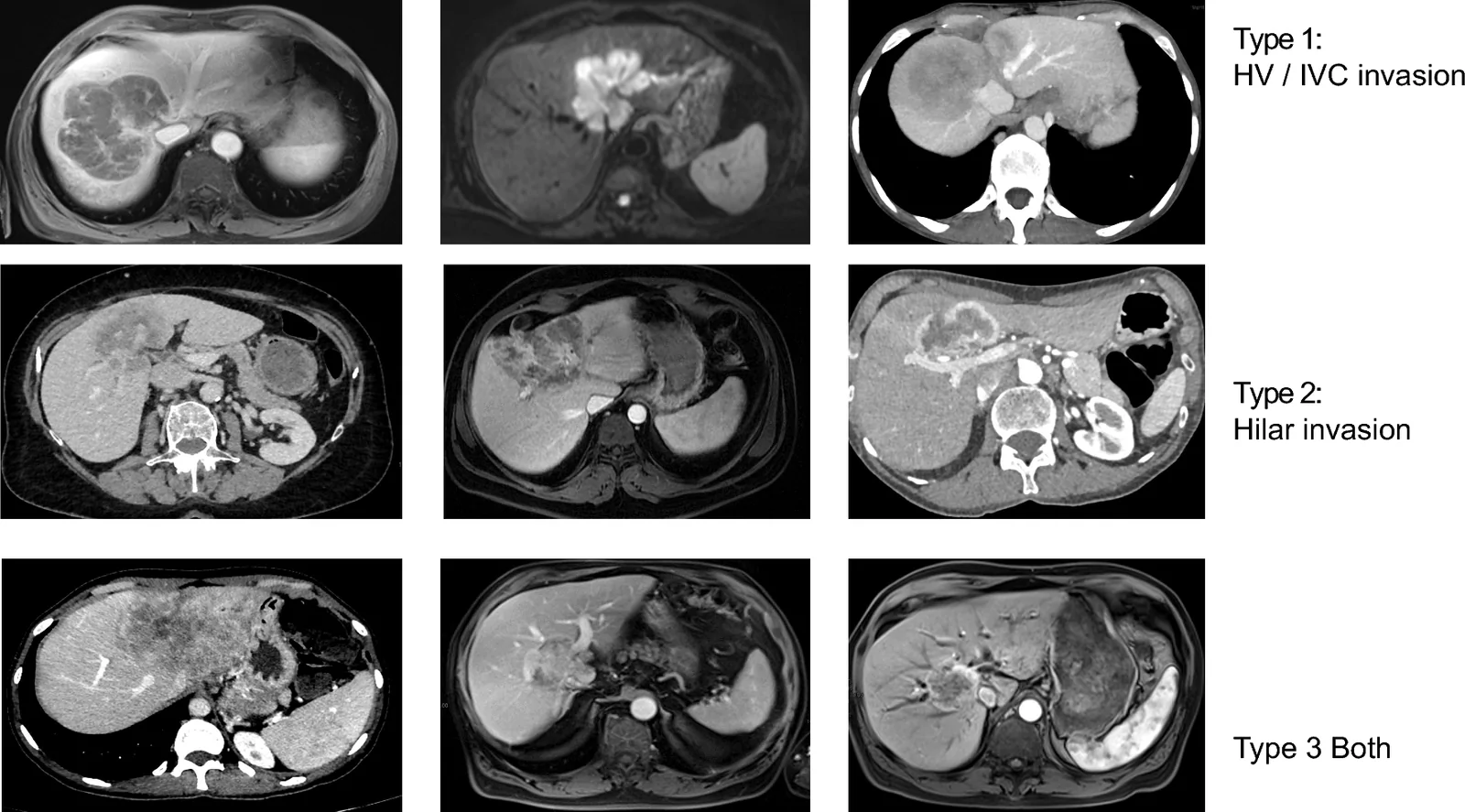
Radiological classification of locally advanced cholangiocarcinomas. *HV* hepatic vein, *IVC* inferior vena cava

This single-center retrospective cohort study analyzed outcomes of patients who underwent extended liver resection for LAICC between 2013 and 2024. LAICC was defined as mass-forming intrahepatic single tumors 5 cm or more in size with venous contact (inferior vena cava (IVC) and/or main hepatic veins) or hepatic hilum contact or both and requiring extended liver resection (five segments or more) (Fig. 1). Liver resections were named according to the New World Nomenclature.^6^ All patients underwent preoperative cross-sectional imaging to evaluate tumor characteristics, vascular invasion patterns. Additional three-dimensional (3D) reconstruction and future liver remnant volume were assessed in all cases. Portal vein embolization was performed when future liver remnant volume was considered insufficient. Preoperative biliary drainage was performed in patients with jaundice. All cases were discussed at our multidisciplinary tumor board. Neoadjuvant chemotherapy or intra-arterial treatments (transarterial chemoembolization, transarterial radioembolization) were administered before referral or according to multidisciplinary tumor board decision.

### Surgical Technique

Procedures were performed by J-shape right subcostal incision with left extension when required. Complete abdominal exploration and intraoperative ultrasound were performed. Once the decision to proceed was confirmed, the liver was mobilized according to the required procedure. Vascular control was performed by the Glissonian approach or intrafascial dissection depending on cases. Parenchymal transection was performed using the cavitron ultrasonic surgical aspirator (CUSA) method under intermittent inflow occlusion or total vascular exclusion (TVE) as required. In cases requiring complex hepatocaval vascular reconstruction, venous bypass and in situ cooling were used. When indicated, resection and replacement of inferior vena cava (IVC) was performed with 16–20-mm ringed polytetrafluoroethylene (PTFE). For hepatic vein (HV) reconstructions, cryopreserved venous graft or PTFE graft was used. Biliary reconstruction consisted in Roux-en-Y hepaticojejunostomy or duct to duct anastomosis in selected cases.^7^ Regional lymphadenectomy was performed in all cases (stations 5, 8, 9, 12, and 13).^8^

### Case Review and Study Outcomes

Data were collected from our prospective database. All imaging studies were individually reviewed by the two main investigators (D.C., E.M.M.). By design, tumors were centrally located or had central extension. Owing to the impact of tumor connections on the nature and magnitude of the required surgery and reconstructions, tumors were classified into three categories: type 1: hepatic vein–IVC contact (HV–IVC); type 2: hilar contact (HIL); type 3: both (HV–IVC–HIL). R1 resection was defined as introperative tumor exposure and/or pathological margin < 1 mm.

Main outcome measures included postoperative morbidity and mortality according to the Clavien classification^9^ and long-term overall and disease-free survival. Post-hepatectomy liver failure was defined according to 50–50 criteria and ISGLS classification for severity.^10,11^ Pattern and management of recurrences were analyzed. Futility was defined as 90-day mortality or recurrence within 6 months of surgery.^12,13^

### Statistical Analysis

Survival was analyzed using Kaplan–Meier method with log-rank tests for comparisons. Overall survival was calculated for the date of surgery until the date of death or last follow-up. Patients who died within 90 days of surgery were excluded from recurrence-free survival analysis. Univariate and multivariate analyses were performed to identify prognostic factors. Statistical significance was set at *p* value of 0.05. Statistical analyses were performed using SPSS software (version 26.0, IBM Corp). The study protocol was designed according to the ethical guidelines of the 1975 Declaration of Helsinki and was approved by the local ethics committee (CPP Bicêtre no. CO 16-006) and the National Commission for Information Technology and Freedom (CNIL no. 1856085).

## Results

### Preoperative Characteristics

Preoperative characteristics are presented in Table 1. Thirty-nine patients (29 women [74%]; median age, 66 years (38–83 years)) underwent ELR for LAICC, all by an open approach. Median tumor size was 7 cm (5–14 cm), with macrovascular and/or hilar invasion in 100% of the cases. Tumor classification was type 1 (HV–IVC) in 8 (20.5%%), type 2 (HIL) in 17 (43.5%%), and type 3 (HV–IVC–HIL) in 14 cases (36%). Vascular invasion consisted in extrinsic encasement in all cases, with no instances of intravascular thrombus. Biliary invasion with obstructive jaundice was present in five patients (12.8%). Neoadjuvant therapies were used in 22 patients (56.5%). Details are presented in Table 1. Treatment response included radiological response in 12 patients (67%), stable disease in 4 (22%), and progression in 2 (11%). Right portal vein embolization was performed in 15 patients (38.5). Preoperative biliary drainage was required in four patients, and preoperative exploratory laparoscopy was performed in four patients.

**Table 1:** Preoperative patient characteristics

Parameter		Value
Age, years^*^		66 (38–83)
Sex, n (%)	Male	10 (25.6%) 29 (74.4)
	Female	
ASA≥2	1 2 3	5 (12.8 %) 23 (59 %) 11(28.2%)
Imaging classification^**^	Type 1 (HHV–IVC) Type 2 (HIL) Type 3 (HV–IVC–HIL)	8 (20.5%) 17 (43.5%) 14 (36%)
Tumor size (cm)^*^		7 (5–14)
Obstructive jaundice		5 (12.8%)
Liver preparation		19 (48.7%)
Portal vein embolization Preoperative biliary drainage		15 (38.5%) 4 (10.5%)
CEA^*^		2 (0.5–14)
CA19.9^*^		44 (2–27,000)
AFP^*^		4 (0–120)
Neoadjuvant chemotherapy		18 (46.15%)
Gemcis Gemcis–Durvalumab Gemox FOLFIRINOX/FOLFOX		6 (33%) 5 (27%) 4 (22%) 3 (16%)
Intravascular neoadjuvant treatment	Y90 radioenmbolization Transarterial chemoembolization	1 (5.5%) 3 (16%)
Radiological response^***^	Response Stability Progression	12 (67%) 4 (22%) 2 (11%)

^*^Values are reported as median (min–max). ^**^ ccording to our classification: type 1: main hepatic vein/IVC invasion*;* type 2: hilar invasion*;* type 3: both. ^***^Response = radiological response to chemotherapy (tumor size reduction)

### Surgical Features

Surgical features are presented in Table 2.

**Table 2:** Surgical features

Parameter	Value
Type of liver resection	
H145678	17 (44%)
H123458	17 (44%)
H45678	4 (10.25%)
H23458	1 (2.5%)
Total vascular exclusion	14 (35%)
Patients without vascular or biliary reconstruction	7 (18%)
Vascular resection	17 (43.5%)
IVC resection With venous bypass Without venous bypass	11 (28%) 8 (72%) 3 (28%)
Hepatic vein reconstruction	7 (18%)
Portal vein resection	4 (14%)
Hepatic artery resection/reconstruction	1 (2%)
Biliary reconstruction	26 (67%)
*Roux en Y*	18 (70%)
*Duct to duct*	8 (30%)
Operative time (min)^*^	420 (250–809)
Blood loss (ml)^*^	500 (0–5600)
Patient trasfused	12 (30%)
Units transfused^*^	0 (0–33)

^*^Values reported as median with range (min–max)

Surgical procedures included six-segment resections in 34 patients (88%) and five-segment resections in 5 patients (12%). Total vascular exclusion was used in 14 patients (35%), including venous bypass in 8 cases (56% of total vascular exclusion cases). Vascular resection was required in 17 patients (43.5%), including IVC resection in 11 (65% of vascular resections), hepatic vein reconstruction in 7 patients (41%), portal vein resection in 4 (23.5%), and hepatic artery reconstruction in 1 (6%). Biliary reconstruction was required in 26 cases (67%). A total of 12 patients (30%) received packed red cells transfusion.

### Morbidity and Mortality

Details about morbidity and mortality are presented in Table 3.

**Table 3:** Postoperative results

Parameter	N (%)
Overall morbidity	22 (56%)
Clavien Dindo IIIb–IV	8 (20%)
90-Day mortality	2 (5.1%)
PHLF (50/50)	7 (17.9%)
PHLF severe^**^	4 (10.3%)
Reoperation	4 (11%)
Percutaneous drainage	4 (11%)
Hospital stay (days)^*^	14 (7–130)

^*^All values reported as median with range (min–max). ^**^According to ISGLS classification. *PHLF* posthepatectomy liver failure

Complications occurred in 22 patients (56%). Severe complications (Clavien–Dindo ≥ 3b) occurred in eight patients (20%) (post-hepatectomy liver failure (50/50 criteria) in seven patients (17.9%), ISGLS B or C in four [10.3%]). Reoperation was required in four patients (11%), and percutaneous drainage in four (11%). Reoperations included two cases of biliary anastomosis leakage and one case each of intra-abdominal sepsis and portal vein thrombectomy. Two patients died within 90 days of surgery (5%) owing to post-hepatectomy liver failure (day 65) in one and portal vein thrombosis in the other (day 10). Median hospital stay was 14 days (7–130 days). Univariate analysis did not identify any statistically significant predictors of severe complications (Suppplementary Table S1).

### Pathological and Oncological Results

Details about pathological data are presented in Table 4.

**Table 4:** Oncological and pathological results

Parameter		N (%)
Median FU (months)^*^		30 (4–144)
Adjuvant chemotherapy^**^		24 (61)
Patients alive at the time of report Without recurrence With recurrence		22 (56%) 8 (20%) 14 (35.9%)
Patients alive at the time of report Without recurrence With recurrence		22 (56%) 8 (20%) 14 (35.9%)
Recurrence		20 (51%)
Treatment of recurrence		
Chemotherapy Surgery		18 (90%) 5 (25%)
Futility^***^		6 (15%)
TNM	< T3 T3–T4	15 (38%) 24 (62%)
N	N+ N0	10 (26%) 29 (74%)
Surgical margins	R1 R0	21 (54%) 18 (46%)
Lymph nodes recovered ≥ 6		25 (64%)

All values reported as median with range (min–max). ^***^Capecitabin. ^***^Death within 90 days or reccurrence within 6 months of surgery

**Fig. 2:**
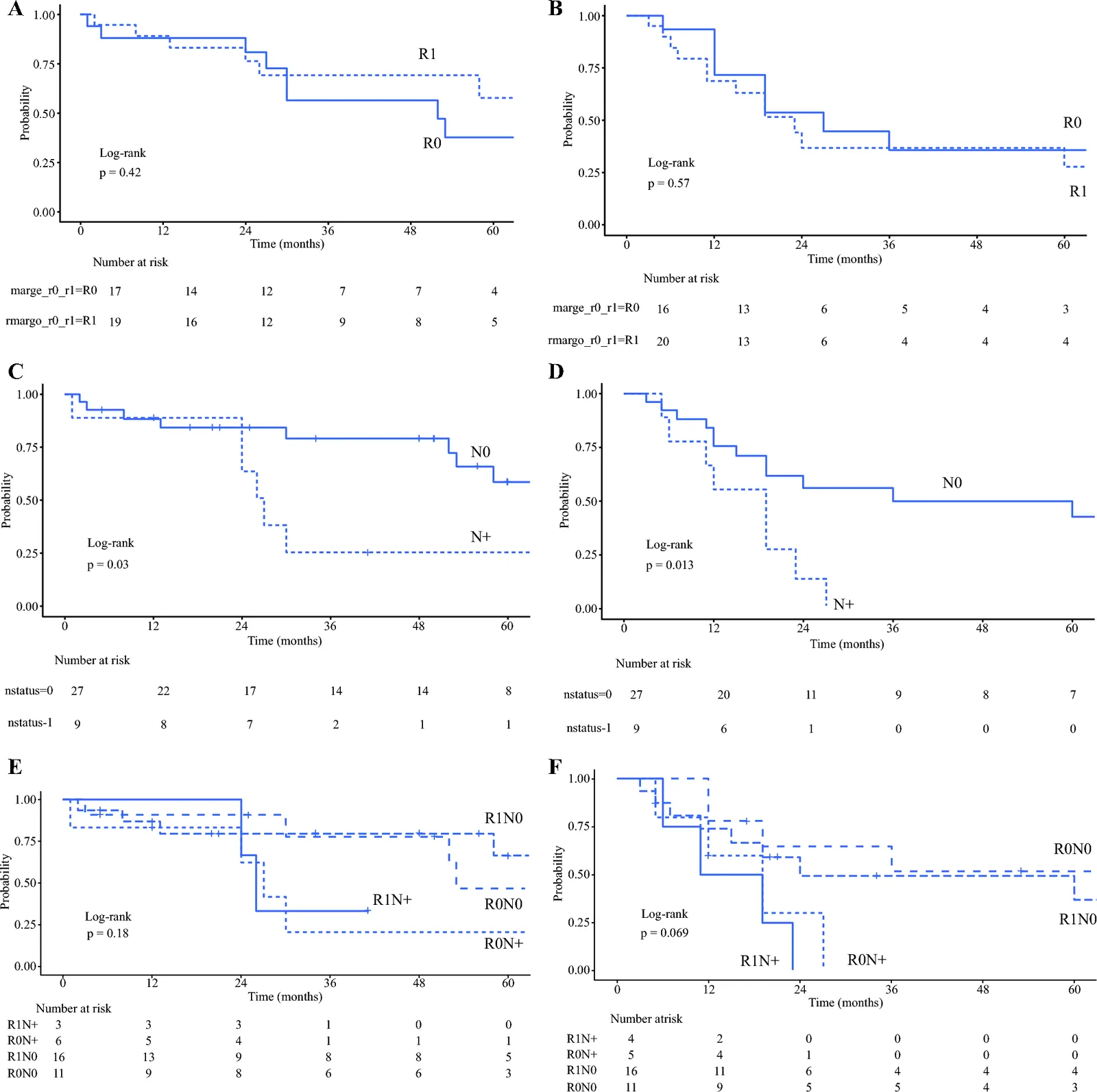
Kaplan–Meier curves for overall (**A**) and recurence-free (**B**) survival between R1 resection R0 resection. Kaplan–Meier curves for overall (**C**) and recurrence-free (**D**) survival between N+ and N0. Kaplan–Meier curve for overall (**E**) and recurrence-free (**F**) survival with matched factor (margin status and N status)

Lymph node metastasis were present in 12 patients (31%), and R1 resection margins in 21 (54%). Adequate lymph node sampling (≥ 6 nodes) was achieved in 25 patients (64%). Adjuvant chemotherapy was administered in 24 patients (61%) (Table 5). Survival data are presented in Table 5. Median follow-up was 30 months. Median overall survival was estimated at 58 months, with 1-, 3-, and 5-year overall survival rates of 88.7%, 63.5%, and 48.3%, respectively. Median recurrrence-free survival was 23 months, with 1-, 3-, and 5-year recurrrence-free survival rates of 70.2%, 36.1%, and 30.9%, respectively. Twenty-two patients (56%) were alive at last follow-up, including 14 (64%) recurrrence-free. Tumor recurrence occurred in 20 patients (51%). Site of recurrences were liver (65%), lung (40%), and peritoneal and lymph node (15% each). Single-site recurrence occurred in half of the cases. Recurrences were treated with chemotherapy in 15 (75%), surgery in 2 (10%), and combined approach in 3 (15%). Overall rate of futility was 15.4% (six patients, including two deaths within 90 days and four recurrences within 6 months). A total of 11 patients were alive more than 5 years after surgery (28%), including 8 without evidence of disease and 3 with controlled disease. Details about these long-term suvivors are presented in Supplementary Table S2. The only factor associated with recurrence was N+ (88.9% versus 44.4%; *p* = 0.020) and adjuvant chemotherapy (73% versus 21.7%; *p* < 0.001). Details are presented in Table 5. Survival analysis demonstrated significantly worse overall and disease-free survival for patients with lymph node metastasis (log-rank test, *p* = 0.03, for OS; log-rank test, *p* = 0.013, *p* = 0.017 for RFS) (Fig. 2). Resection margin status (R0/R1) did not significantly impact survival outcomes (log-rank test, *p* = 0.58, for overall survival (OS); log-rank test, *p* = 0.57, for recurrrence-free survival; Fig. 2). Figure 2 shows survival curves combining lymph node and margin status.

**Table 5:** Univariate analysis for predictive factors of recurrence

Parameter	No recurrence (n = 16)	Recurrence (n = 20)	*p*-Value
Age, years	64	63.5	0.583
Sex, n (%)			0.439
M			
	3 (30%)	6 (60%)	
F	13 (44.8%)	14 (48.3%)	
ASA ≥ 2	5 (50%)	50 (50%)	0.677
Macrovascular invasion	9 (45%)	11 (55%)	0.940
Tumor size (cm)	7	7	0.987
Biliary drainage	1 (50%)	1 (50%)	0.871
Portal vein embolization	6 (46.2%)	7 (53.8%)	0.877
Neoadjuvant chemotherapy	6 (37.5%)	10 (62.5%)	0.453
CEA^*^	1.95	2.00	0.304
CA19.9^*^	36.4	66	0.142
AFP^*^	4.25	4	0.489
Type of liver resection			0.708
H123458	7 (41.2%)	10 (58.8%)	
H23458	1 (100%)	0 (0%)	
H45678	2 (50%)	2 (50%)	
H145678	6 (42.9%)	8 (57.1%)	
Hepatic hilum invasion	9 (39.1%)	14 (60.9%)	0.393
Vascular resection	7 (44.4%)	8 (53.3%)	0.821
IVC resection	4 (44.4%)	5 (55.6%)	1
Bilioenteric anastomosis	11 (68.8%)	5 (31.3%)	0.119
Operative time (min)^*^	403	410	0.956
Transfusion (units)^*^	0	0	0.218
Overall morbidity	11 (57.9%)	8 (42.1%)	0.086
TNM			0.112
< T3	9 (60%)	6 (40%)	
T3–T4	7 (33.3%)	14 (66.7%)	
Surgical margin			0.549
R0	8 (50%)	8 (50%)	
R1	8 (40%)	12 (60%)	
**N status**			**0.020**
**N0**	**15 (55.6%)**	**12 (44.4%)**	
**N+**	**1 (11.1%)**	**8 (88.9%)**	
Reponse to neodjuvant chemotherapy	4 (30.8%)	9 (69.2%)	0.141

^*^All values reported as median with range (min–max)

## Discussion

ICC is a severe disease for which surgical resection represents the only prospect for long-term survival or cure. The recognized oncologic factors for survival after surgery include lymph node invasion and margin status.^2^ According to most guidelines, indications for surgery include the possibility of R0 resection and sufficient liver remnant.^2^ However, owing to its initial indolent progression, ICC is often diagnosed at an advanced stage, including large tumor size and vascular or biliary connections or both, raising the concept of LAICC. This results in a conflicting dilemma where the prospect of anavoidable R1 resection due to contact with vital vascular structures often leads to not considering resection. We have chosen an aggressive surgical approach in such cases, and our study supports the fact that anticipated R1 by necessity should not be considered a contraindication to resection. Indeed, our series shows that overall and recurrence-free survival were similar in R0 and R1 resections, regardless of nodal status. This finding, which contrasts with established oncologic surgical principles, has nonetheless been previously reported in the literature and may be explained by emerging evidence suggesting that microscopic margin status may not always independently influence long-term outcomes. For instance, in the study by Endo et al., it was shown that, as the tumor burden score increases, the prognostic impact of margin status progressively decreases, becoming nonsignificant when the score exceeds 6.^14^ Moreover, as reported in a French multicenter study published in 2011, the presence of lymph node metastases overrides the prognostic value of surgical margin status, with nodal involvement emerging as the dominant determinant of long-term outcomes.^15^ Of course, R0 resection should be the goal, but in locally advanced disease, R1 seems to be acceptable provided low mortality and morbidity are achieved.

By contrast, N status remains the main prognosticator for recurrence or survival. In our series, 31% of the patients were N+ and 100% of them had recurrence within 2 years. However, 5-year survival was achieved in 25% of N+ patients thanks to treatment of recurrences. This may justify resections even in the presence of positive locoregional lymph nodes, again provided low mortality and morbidity are achieved.

Despite meticulous lymph node dissection to obtain ≥ 6 LN, this was achieved in only 64% of the cases. This may be a technical issue, although we have a standardized approach to perihilar lymphadenectomy. Other possibilities may include insufficient surgeon–pathologist communication or insufficient identification on the specimen. The main reason for lymphadenctomy is staging, although an intrinsic therapeutic role has been suggested.^16^ The main issue with low lymph node retrieval is understaged nodal status when the number of retrieved LN is insufficient.

We defined locally advanced ICC as single intrahepatic tumors 5 cm or more in size with venous contact (IVC and/or main hepatic veins) or hepatic hilum contact or both and requiring extended liver resection (five segments or more). Consequently, this study reports only complex procedures including extended parenchymal removal and vascular and/or biliary resections in 32 (82%) of the cases. Only seven cases (18%) did not require vascular or biliary reconstruction. Patient preparation was performed according to individual cases, including portal vein embolization in case of insufficient liver remnant (38% of cases, only for right-sided resections) and/or biliary decompression in case of obstructive jaudice (10%). Of note, despite hilar contact in 80% of the cases, obstructive jaundice was present in only 12% of cases, by contrast with hilar cholangiocarcinoma where intraluminal growth is the initial pattern and jaundice the common feature. Procedures were performed by highly trained surgeons (two attending surgeons in most cases) and anesthesiologists, and postoperative care was precise including initial stay in the intensive care unit (ICU) for 1 or 2 days and monitoring for complications. Such perioperative procedures allowed a low mortality, severe morbidity (Clavien > IIIa), and futility rates of 5%, 11%, and 15%, respectively. In a recent meta-analysis including close to 20,000 cases of ICC, mortality for extended resection was 11.5%, against which our results compare favorably.^17^

Of note, unlike previous studies, in our series, vascular resections and/or biliary reconstructions did not result in a significant increase in postoperative morbidity^18^ (Supplementary Table S1). Patients who underwent resection with vascular involvement had complication rates comparable to those without vascular procedures. Experience in liver transplantation is probably an advantage for that matter.

Our series provides compelling evidence that even patients with LAICC can achieve long-term survival when managed with an aggressive strategy. Among the 11 patients (28%) surviving beyond 5 years (Supplementary Table S2), 6 patients remained disease-free after extended hepatectomy despite the high tumour burden and frequent need for vascular and/or biliary reconstruction.

A particularly informative subgroup is represented by patients who recurred but nevertheless remained long-term survivors. These cases illustrate the value of the type, location, and active management of recurrence, including repeat hepatectomy and modern systemic therapy. For example, three patients in our cohort underwent repeat hepatectomy for intrahepatic recurrence, with one subsequently undergoing salvage liver transplantation, achieving more than 5 years of survival with controlled extrahepatic disease. Another patient, with R0/N+ disease, experienced both hepatic and nodal recurrence but achieved prolonged metabolic and morphologic remission following a combination of repeat hepatectomy, systemic therapy, and targeted treatment with an IDH1 inhibitor.^19^

The use of neoadjuvant treatment is logical in these locally advanced tumor with a downstaging intent prior to surgery. However, this has not been supported with strong evidence so far with small, mostly phase II studies.^20^ Two randomized still unpublished studies were presented this year at major meetings showing promising results with neoadjuvant chemotherapy with or without immunotherapy.^21^ For many years, first-line standard of care for advanced disease has been the combination of gemcitabin and cisplatin (GemCis).^22^ More recently, the addition of immunotherapy (durvalumab) to GemCis has proven beneficial in terms of overall and disease-free survival as well as response rate.^23^ In our study, 67% of the patients received preoperative treatments including chemotherapy in 21% and transarterial chemoembolization or radioembolization (all transarterial treatments were administered before referral to our care). Some degree of radiological response to neoadjuvant therapies was observed in 67% of the cases. This did not influence oncologic outcomes, and no sound conclusion can be drawn from these heterogeneous data. In our most recent patients, GemCis + Durvalumab was used with an intent of downstaging. Adjuvant treatments are also desirable, especially in pN+ patients. To date, only adjuvant capecitabine has proven beneficial in biliary tract cancer, and 61% of our patients received it accordingly.^24^ Patients who received adjuvant chemotherapy have paradoxically a higher recurrence rate, but this is obviously related to a selection bias. Recent advances include the exponential progress based on tumor sequencing, specifically in ICC, IDH1 mutations, and FGFR2 rearrangements for which targeted therapies are increasingly available.^19,25,26^ These treatments are only indicated as second-line treatments, but it is possible that, in the near future, such targeted therapies may be proposed at an earlier stage, even preoperatively, on the basis of sequencing from biopsy samples or circulating tumor DNA. Liquid biopsies are increasingly being used for prognostic reasons but also to propose more personalized therapy. Intensified staging with ^18^F-fluorodeoxyglucose (FDG) positron emission tomography (PET)/computed tomography (CT) and endoscopic ultrasound (EUS)-guided lymph node biopsy may improve patient selection for neoadjuvant treatment. Given the prognostic significance of nodal involvement in intra-hepatic cholangiocarcinoma (iCCA), integration of these modalities may improve oncological outcomes.^27,28^

This study has several limitations. First, its retrospective, single-center design inherently carries a risk of selection bias and limits the sample size. These limitations have a significant impact on the robustness of the results; however, the cases analyzed in this series are uncommon owing to the high tumor burden of the patients and the surgical complexity involved. The granularity of the dataset with each case being individually and meticulously reviewed represents a strength of the study, allowing for more detailed clinical insights than might be possible in larger, multicenter cohorts. Second, perioperative neoadjuvant and adjuvant treatments were heterogeneous and not standardized owing to the numerous evolutions that occurred during the last 10 years, which is the span of this study. Third, owing to the small sample size, the statistical methodology is also limited and performed according to the study design. Fourth, the present study comes from a high-volume tertiary hepato-pancreato-biliary (HPB) center with advanced expertise in major liver surgery and liver transplantation; therefore, the reproducibility in terms of risk and outcomes in lower-volume centers or less specialized settings may be limited.^29^ Also several patients were referred after previous treatment had been administered.

## Conclusions

Locally advanced ICC requiring extended liver resection remains a formidable surgical challenge. Our study supports an aggressive surgical approach allowing close to 50% overall 5-year survival. R1 resection by necessity has no prognostic impact and should not be considered a contraindication to surgery. Nodal status is the main driver of prognosis. While tumor biology may outweigh technical factors in determining outcomes, individual patients should not be denied a chance of long-term survival when surgery is possible at low risk in a highly experienced environment.

## Supplementary Information

Below is the link to the electronic supplementary material.

Supplementary file1 (DOCX 22 kb)

Supplementary file2 (DOCX 17 kb)

## Data Availability

The data are available upon request from the corresponding author.
