# Supplementary material for: Is R1 Extended Liver Resection for Locally Advanced Intrahepatic Cholangiocarcinoma Justified? Nodal Status Not Margin Drives Prognosis
Source: Ann Surg Oncol. 2026 Jun 29;33(12):9991–10000. doi: 10.1245/s10434-026-20034-3 (PMC13675155; doi:10.1245/s10434-026-20034-3)
Supplement: Supplementary file 1 [file 10434_2026_20034_MOESM1_ESM.docx]

**Table S1.** Predictive factor of severe mordibity

| **Parameter** | | **No severe morbidity (CD≤3a) (n = 17)** | **Severe morbidity (CD>3a) (n=5)** | **p value** |
| --- | --- | --- | --- | --- |
| Age, years* |  | 65(38-83) | 67(39-74) | 0.850 |
| Sex, n (%)  Male  Female |  | 7 (87.5%)  10 (71.4%) | 1 (12.5%)  4 (28.6%) | 0.387 |
| ASA ≥2 |  | 6(85.7%) | 1(14.3%) | 0.519 |
| FLR/BW* |  | 0.800 | 0.820 | 0.893 |
| Macrovascular Invasion |  | 11(73.3%) | 4(26.7%) | 0.519 |
| Tumor size (cm)* |  | 7(5-14) | 7(5-14) | 0.468 |
| Biliary drainage |  | 4(80%) | 1(20%) | 0.442 |
| Embolisation Portal |  | 7(70%) | 3(30%) | 0.457 |
| Neoadjuvant chemotherapy |  | 8(66.7%) | 4(33.3%) | 0.193 |
| Type of liver resection  H123458  H23458  H45678  H145678 |  | 6(85.7%)  1(100%)  1(50%)  9(75%) | 1(14.3%)  0(0%)  1(50%)  3(25%) | 0.691 |
| Hepatic hilum invasion |  | 11(68.8%) | 5(31.3%) | 0.119 |
| Bilioenteric anastomosis |  | 11(68.8%) | 5(31.3%) | 0.119 |
| Operative time* |  | 363(250-675) | 480 (330-800) | 0.084 |
| Trasfusion |  | 0(0-18) | 3(0-33) | 0.096 |
| TVE |  | 6(66.7%) | 3(33.3%) | 0.323 |
| Venous Bypass |  | 6(85.7%) | 1(14.3%) | 0.519 |
| In situ cooling |  | 6(75%) | 2(25%) | 0.848 |
| Vascular resection |  | 9(75%) | 3(25%) | 0.781 |
| IVC resection |  | 6(75%) | 2(25%) | 0.848 |
|  |  |  |  |  |

*All values are reported with median and range (range)
