# Supplementary material for: Is R1 Extended Liver Resection for Locally Advanced Intrahepatic Cholangiocarcinoma Justified? Nodal Status Not Margin Drives Prognosis
Source: Ann Surg Oncol. 2026 Jun 29;33(12):9991–10000. doi: 10.1245/s10434-026-20034-3 (PMC13675155; doi:10.1245/s10434-026-20034-3)
Supplement: Supplementary file 2 [file 10434_2026_20034_MOESM2_ESM.docx]

|  | Age /sex | Type*/Size** | Neoadjuvant/  Response | Resection | R/N | Adjuvant | Recurrence/  Site | Treatment | Time since surgery |
| --- | --- | --- | --- | --- | --- | --- | --- | --- | --- |
| **Without disease** |  |  |  |  |  |  |  |  |  |
| 1 | 73/M | 3/7 | No | H123458 | R1/N0 | No | No |  | 108 months |
| 2 | 51/F | 1/9 | No | H123458 HV IVC | R1/N0 | No | No |  | 96 months |
| 3 | 78/F | 3/6 | No | H123458 | R1/N0 | No | No |  | 84 months |
| 4 | 51/M | 2/10 | No | H123458 B | R1/N0 | Yes | Yes/liver | Repeat heptectomy | 103 months |
| 5 | 62/F | 1/5 | No | H123458 | R1/N0 | Yes | No |  | 144 months |
| 6 | 70/F | 2/5 | No | H145678 B PV | R0/N0 | No | No |  | 84 months |
| 7 | 47/F | 2/5 | No | H145678 B | R0/N0 | No | No |  | 96 months  (died other cancer) |
| 8 | 38/F | 3/6 | Yes | H123458 B A | R0/N+ | Yes | Yes/Liver and lymph node | Repeat hepatectomy, Chemo, Anti IDH1 | 91 months in metabolic and morphologic remission without treatment |
| **With disease** |  |  |  |  |  |  |  |  |  |
| 1 | 66/F | 3/6 | 0 | H123458 | R0/N0 | Chemo | Yes/liver and lung | Chemo | 60 months stable on chemo |
| 2 | 61/F | 2/8 | 0 | H145678 B | R1/N0 | Chemo | Yes/Liver | Repeat hepatectomy, Liver transplant | 64 months pulmonary mets stable on chemotherapy |
| 3 | 69/F | 2/7 | Yes | H45678 | R1/N0 | Yes | Yes/lymph node | Chemo, Surgery | 65 months stable on chemo |

*based on vasculobiliary connections (see figure 1), **cm,

Supplementary table : Long term survivors (> 5 years) after extended resection for locally advanced intrahepatic cholangiocarcinoma
